# High-Definition Videobronchoscopy for the Diagnosis of Airway Involvement in Sarcoidosis: The Enhance Sarcoidosis Multicenter Study

**DOI:** 10.1016/j.chest.2023.04.034

**Published:** 2023-04-28

**Authors:** Vanina Livi, Ilya Sivokozov, Jouke T. Annema, Piero Candoli, Igor Vasilev, Tess Kramer, Marco Ferrari, Karan Madan, David Fielding, Septimiu Murgu, Alessandra Cancellieri, Lyudmila A. Semyonova, Mariangela Puci, Giovanni Sotgiu, Rocco Trisolini

**Affiliations:** a Division of Interventional Pulmonology, Department of Neurosciences, Sense Organs and Thorax, Fondazione Policlinico Universitario A. Gemelli IRCCS, Rome; b Pathology Unit, Fondazione Policlinico Universitario A. Gemelli IRCCS, Rome; c Department of Cardiovascular and Pulmonary Sciences, Catholic University of the Sacred Hearth, Rome; d Interventional Pulmonology Unit, IRCCS Azienda Ospedaliero-Universitaria di Bologna, Bologna; e Clinical Epidemiology and Medical Statistics Unit, Department of Medicine, Surgery and Pharmacy, University of Sassari, Sassari, Italy; f Endoscopy Department, Central TB Research Institute, Moscow, Russia; g Department of Pathomorphology, Cell Biology and Biochemistry, Central TB Research Institute, Moscow, Russia; h State Research Institute of Phtisiopulmonology, St. Petersburg, Russia; i Department of Respiratory Medicine, Amsterdam UMC, Amsterdam, The Netherlands; j Department of Pulmonary, Critical Care and Sleep Medicine, All India Institute of Medical Sciences, New Delhi, India; k Department of Thoracic Medicine, The Royal Brisbane & Women’s Hospital, Brisbane, QLD, Australia; l UQ Centre for Clinical Research, Faculty of Medicine, The University of Queensland, Brisbane, QLD, Australia; m Section of Pulmonary and Critical Care Medicine/Interventional Pulmonology, The University of Chicago, Chicago, IL

**Keywords:** CT scan, endobronchial biopsy, endobronchial ultrasound, granuloma, high-definition videobronchoscopy, sarcoidosis

## Abstract

**Background:**

The ability of high-definition (HD) videobronchoscopy to detect airway involvement in sarcoidosis has not been evaluated previously.

**Research Question:**

What is the role of HD videobronchoscopy in the identification of sarcoidosis-associated airway abnormalities (AAs)? What are the patterns of AAs more commonly observed and more frequently associated with the detection of granulomas in endobronchial biopsy (EBB)?

**Study Design and Methods:**

In this prospective international multicenter cohort study, consecutive patients with suspected sarcoidosis underwent airway inspection with an HD videobronchoscope and EBB using a standardized workflow. AAs were classified according to six patterns defined a priori: nodularity, cobblestoning, thickening, plaque, increased vascularity, and miscellaneous. We assessed diagnostic yield of EBB, prevalence of AAs, and interobserver agreement for different patterns of AAs.

**Results:**

AAs were identified in 64 of 134 patients with sarcoidosis (47.8%), with nodularity (n = 23 [17.2%]), plaque (n = 19 [14.2%]), and increased vascularity (n = 19 [14.2%]) being the most prevalent. The diagnostic yield of EBB was 36.6%. AAs were significantly more prevalent in patients with than in those without nonnecrotizing granulomas on EBB (67.4% vs 36.5%; *P* = .001). Likewise, parenchymal disease on CT scan imaging was significantly more common in patients with than in those without nonnecrotizing granulomas on EBB (79.6% vs 54.1%; *P* = .003). On a per-lesion analysis, nonnecrotizing granulomas were seen especially in EBB samples obtained from areas of cobblestoning (9/10 [90%]) and nodularity (17/29 [58.6%]). The overall diagnostic yield of random EBB was low (31/134 [23.1%]). The interobserver agreement for the different patterns of AA was fair (Fleiss κ *=* 0.34).

**Interpretation:**

In a population with a large prevalence of White Europeans, HD videobronchoscopy detected AAs in approximately one-half of patients with sarcoidosis. The diagnostic yield of EBB was higher in patients with parenchymal involvement on CT scan imaging and in those with AAs, especially if manifesting as cobblestoning and nodularity.

**Trial Registry:**

ClinicalTrials.gov; No.: NCT4743596; URL: www.clinicaltrials.gov

**Take-home Points**

**Study Question:** What is the role of high-definition (HD) videobronchoscopy in the identification of sarcoidosis-associated airway abnormalities (AAs)?

**Results:** HD videobronchoscopy identified AAs in approximately one-half of patients with sarcoidosis. Patients with AAs and those with parenchymal disease on CT scan imaging were significantly more likely to have nonnecrotizing granulomas identified in endobronchial biopsy (EBB) samples. The diagnostic yield of EBB samples was highest in patients with cobblestoning and nodularity patterns and lowest in patients with increased vascularity.

**Interpretation:** Given its simple technical performance, low morbidity, and nonnegligible diagnostic yield for the detection of granulomas, we suggest that EBB be used in patients with clinicoradiologic suspicion of sarcoidosis featuring any AA other than the increased vascularity pattern.

Endobronchial biopsy (EBB) has been used since 1941 for diagnosing sarcoidosis.^1^, ^2^, ^3^, ^4^ It is easy to perform, safe, and cheap, and it increases the overall granuloma detection rate when coupled with other bronchoscopic sampling methods.^5^, ^6^, ^7^, ^8^, ^9^, ^10^, ^11^, ^12^, ^13^, ^14^, ^15^ Unfortunately, its diagnostic yield has shown wide variability (5%-71%) in different populations,^5^, ^6^, ^7^, ^8^, ^9^, ^10^, ^11^, ^12^, ^13^, ^14^, ^15^, ^16^, ^17^, ^18^, ^19^, ^20^, ^21^ partly explained by methodologic limitations (eg, retrospective design, small sample size, EBB not performed in the entire sarcoidosis population receiving a diagnosis during the study period, and lack of description of the prevalence and pattern of airway abnormalities [AAs]).^5^, ^6^, ^7^, ^8^, ^9^, ^10^, ^11^, ^12^, ^13^, ^14^, ^15^, ^16^, ^17^, ^18^, ^19^, ^20^, ^21^ However, some clinical predictors of EBB success generally are agreed on. Specifically, patients with sarcoidosis with endoscopically visible AAs seem more likely to have granulomas demonstrated by EBB in most studies.^5^, ^6^, ^7^, ^8^, ^9^, ^10^^,^^16^ Moreover, the EBB yield was consistently higher in US populations, which are characterized by a higher prevalence of African American patients.^5^, ^6^, ^7^

In some geographic settings such as Europe, the prevalence of sarcoidosis-associated endobronchial abnormalities was found to be low in studies performed with white light bronchoscopy or standard definition videobronchoscopy.^15^^,^^16^ An imaging method that helped to detect AAs when they are present but not plainly visible with conventional bronchoscopic imaging tools theoretically could help to increase the diagnostic yield of EBB. The recent introduction of high-definition (HD) videobronchoscopes, which provide high-resolution images and real-time optical or digital enhancement techniques, might help to detect subtle sarcoidosis-associated AAs and to characterize various patterns of airway involvement. The present study was aimed at evaluating the diagnostic yield of EBB guided by HD videobronchoscopy, the prevalence of different patterns of AAs, and the detection rate of granulomas associated with different patterns of AAs in patients with suspected sarcoidosis.

## Study Design and Methods

### Study Design and Patients

This international multicenter prospective cohort study was approved by the ethics committee of each participating center (Rome, Amsterdam, Moscow, Bologna, and Saint Petersburg). Patients demonstrating clinical and radiologic suspicion of sarcoidosis were recruited consecutively between April 1, 2021, and March 31, 2022. Inclusion criteria were as follows: (1) indication for pathologic confirmation of a clinical and radiologic (CT scan imaging) suspicion of sarcoidosis, (2) age older than 18 years, (3) American Society of Anesthesiologists score of 1 to 3. Exclusion criteria were: (1) inability or unwillingness to consent to participate, (2) steroid therapy (at least 1 week) in the 2 months preceding bronchoscopy, (3) pregnancy, (4) uncontrolled coagulopathy, and (5) contraindication to the temporary interruption of anticoagulants or antiplatelet drugs, except aspirin.

### Procedure

**Figure 1:**
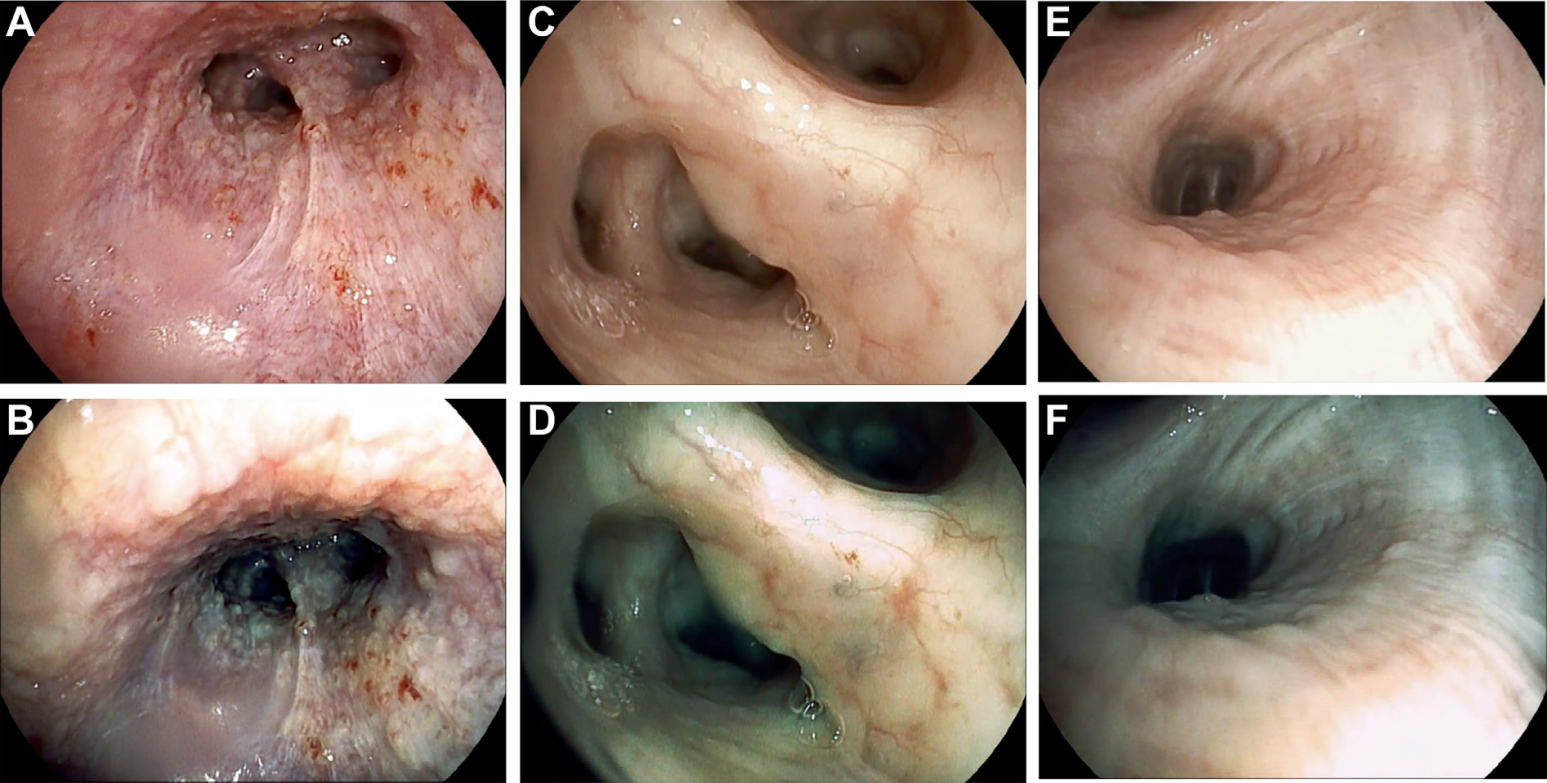
A-F, Endoscopic (i-scan 1 [surface enhancement] and i-scan 2 [surface plus tone enhancement]) images of the following airway abnormality patterns: cobblestoning (A-B), thickening (C-D), and nodularity (E-F).

**Figure 2:**
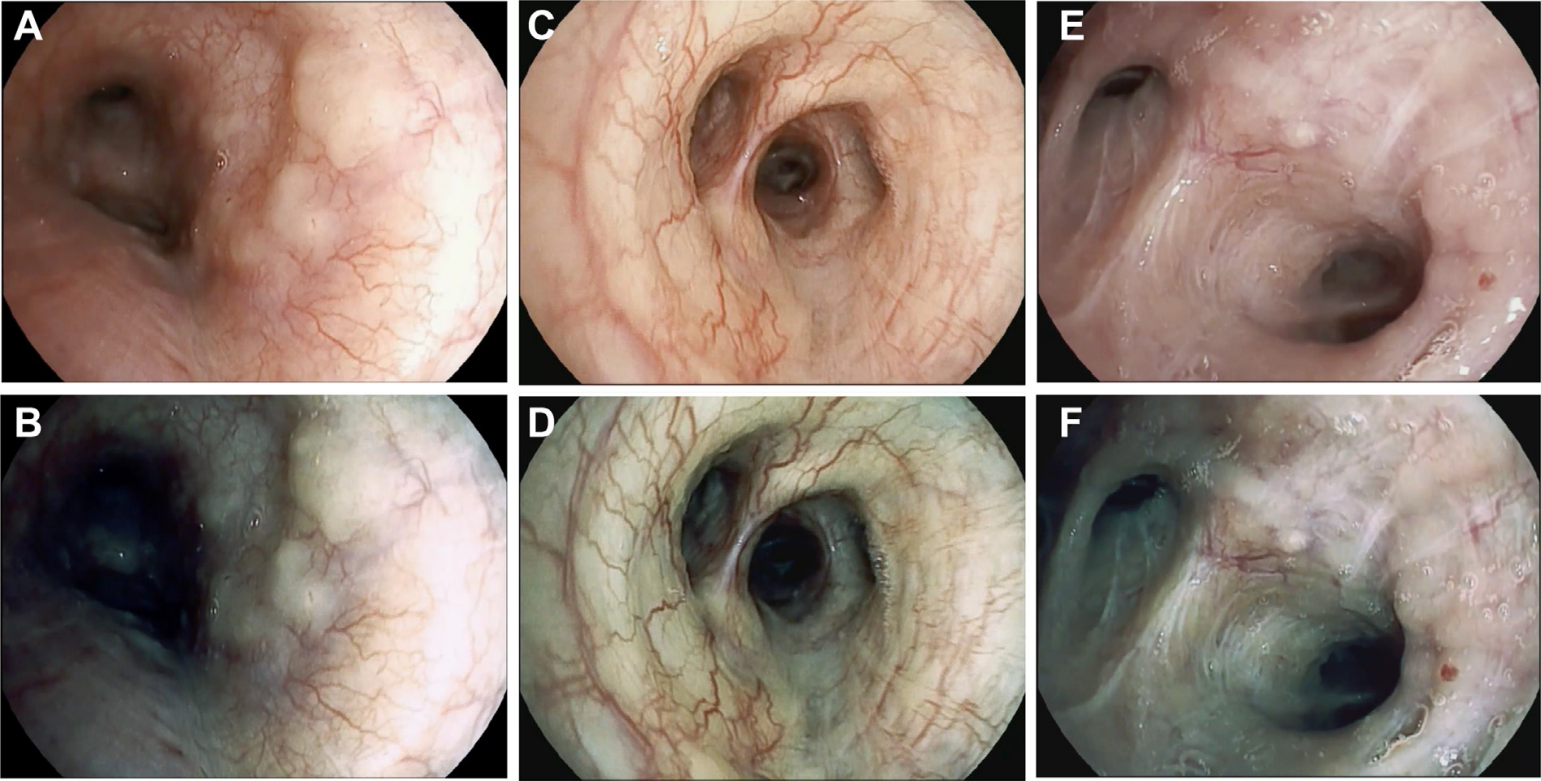
A-F, Endoscopic (i-scan 1 [surface enhancement] and i-scan 2 [surface plus tone enhancement]) images of the following airway abnormality patterns: plaque (A-B), increased vascularity (C-D), and miscellaneous (E-F).

**Figure 3:**
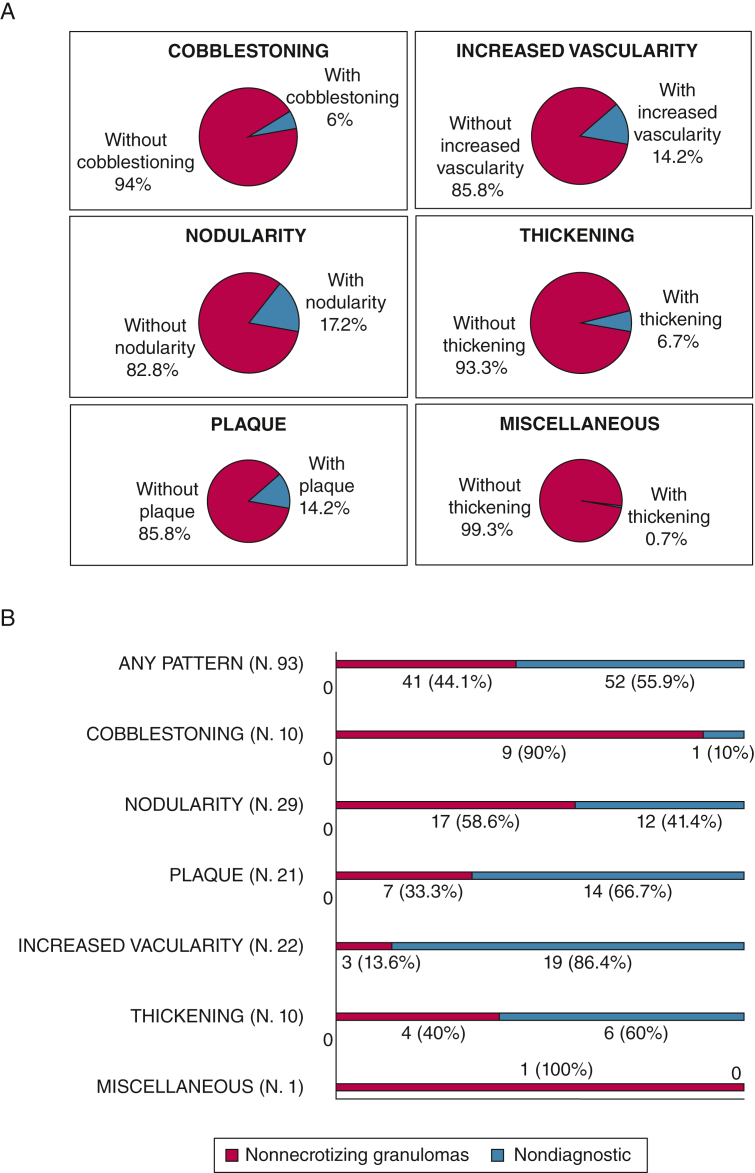
A, B, Pie charts showing the prevalence of different patterns of airway abnormalities (per-patient analysis, n = 134) (A) and bar graphs showing diagnostic yield for the detection of granulomas of different patterns of airway abnormality (per-lesion analysis, n = 93) (B).

Bronchoscopies were performed in each center with the same equipment (Pentax EB15-J10 HD videobronchoscopes coupled with the Defina EPK 3000 processor), identical software settings (e-Appendix 1), and a standardized workflow, as described herein. Briefly, the airways of each patient enrolled first were inspected with the HD videobronchoscope. When AAs were identified, they were characterized further with the surface enhancement (surface enhancement,i-scan 1) and surface plus tone enhancement (tone enhancement mode c,i-scan 2) digital image enhancement technologies, which are integrated in the previously mentioned equipment. Every AA was recorded in a 10- to 15-s video and was classified by the bronchoscopist performing the procedure according to one of six patterns, predefined by the investigators in a meeting held before the study commenced as follows: (1) cobblestoning, ie, the presence of nodules coalescing in a larger infiltrative area (Fig 1A-B); (2) thickening, ie, the presence of smooth or irregular swelling of the mucosa involving a carina (Fig 1C-D); (3) nodularity, ie, the presence of small, discrete nodules (Fig 1E-F); (4) plaque, ie, the presence of infiltrative, raised, or flat whitish or yellow discrete area(s) not involving a carina (Fig 2A-B); (5) increased vascularity, ie, the presence of marked hyperemia or marked increase of submucosal vessel visibility, diffuse or regional (Fig 2C-D); and (6) miscellaneous, ie, the presence of an AA not well described by one of the previous patterns (Fig 2E-F).

EBBs were performed with standard 1.9-mm cupped flexible forceps according to a prespecified protocol: in patients with no endobronchial abnormalities, six biopsy samples were obtained randomly from normal-appearing airway mucosa, whereas in those with AAs, four biopsy samples were obtained from abnormal-appearing areas and two biopsy samples were obtained from normally appearing airway mucosa. Random biopsy samples were obtained from both the right and left airways, but the precise location was left to the discretion of the operators. In patients with different patterns of AAs or different areas of AA belonging to the same pattern, the decision to sample one or more such areas was left to the discretion of the operator. Biopsy samples from each area of abnormal-appearing airway mucosa as well as the biopsies from normal areas were collected in separate vials and were analyzed separately. All the biopsy samples were reviewed by a single pathologist, who was the assigned physician at the time of bronchoscopic sampling and was masked to the endoscopic findings.

Ancillary sampling procedures other than EBB (eg, endobronchial ultrasound-guided transbronchial needle aspiration [EBUS-TBNA], BAL, transbronchial lung biopsy, and transbronchial lung cryobiopsy) were allowed and were performed in the same session at the discretion of the operator, after the endobronchial inspection and biopsy. A sample for microbiologic testing (culture for bacteria and fungi, polymerase chain reaction, and culture for mycobacteria) always was obtained to rule out an infectious cause.

The final diagnosis of sarcoidosis was assigned after review of the clinical, radiologic, microbiologic, and pathologic information by the respiratory physicians running the outpatient sarcoidosis clinic at each enrolling center, after board discussion for low-confidence diagnostic cases. The interventional pulmonologists performing the study procedures were not involved in the final diagnosis assignment process.^22^ When the invasive sampling procedures failed to demonstrate a nonnecrotizing granulomatous inflammation in at least one organ, a clinicoradiologic re-evaluation at 6 months was required to confirm or exclude a provisional diagnosis of sarcoidosis. The follow-up was carried out in sarcoidosis clinics at each enrolled center and took into account the results of follow-up tests (including PET scans and biomarkers) performed in the 6 months after the initial bronchoscopy.

### Study End Points

The primary outcome of the study was the diagnostic yield of the endobronchial biopsy for the detection of granulomas, on a per-patient basis. Secondary outcomes were: (1) overall prevalence of AAs identified with HD videobronchoscopy in patients with sarcoidosis; (2) prevalence of the different patterns of AAs in patients with sarcoidosis; (3) specificity for the detection of granulomas for each predefined pattern of AA; (4) diagnostic yield for the detection of endobronchial granulomas according to sex, ethnicity, history of malignancy, sarcoidosis stage (I-IV), presence vs absence of endobronchial abnormalities at HD bronchoscopy, and pattern of AA detected during HD bronchoscopy; and (5) interobserver agreement for the categorization of the AAs according to the six predefined patterns. For the assessment of the interobserver agreement, the videos referring to each endobronchial abnormality submitted to biopsy examination were evaluated and were classified independently by three interventional pulmonologists (K. M., S. M., and D. F.) masked to the clinical, radiologic, and pathologic data and working on continents different from the one where the study was carried out.

### Statistical Analysis

Categorical variables are described as absolute and relative (percentage) frequencies, whereas quantitative variables are reported as mean ± SD or as median (interquartile range) if normally or nonnormally distributed, based on the Shapiro-Wilks test. Qualitative variables are compared with the χ^2^ or Fisher exact test, as appropriate. The Student *t* test or Mann-Whitney *U* test were used for the comparison of normally or nonnormally distributed quantitative variables, respectively. Logistic regression modelling was used to assess independent factors associated with a positive yield. Fleiss’ κ statistic was used for the analysis of interobserver agreement (values of ≤ 0 indicate poor agreement, values of 0.01-0.20 indicate slight agreement, values of 0.21-0.40 indicate fair agreement, values of 0.41-0.60 indicate moderate agreement, values of 0.61-0.80 indicate substantial agreement, and values of 0.81-1.00 indicate almost perfect agreement). A *P* value of ≤ .05 was considered statistically significant. All the statistical computations were performed with Stata version 17 statistical software (StataCorp).

## Results

During the study period, 172 patients were evaluated for eligibility and 152 were enrolled (e-Fig 1). Table 1 shows the main demographic and clinical characteristics of the 134 patients who finally received a diagnosis of sarcoidosis. The median age was 52 years (interquartile range, 35-60 years), and female participants represented 56.7% of the population. Most patients (n = 123 [93.3%]) were White and demonstrated stage I (n = 49 [36.6%]) or stage II (n = 79 [58.9%]) disease on chest CT scan imaging. Ten patients (7.5%) had a history of malignancy diagnosed within 5 years. The final diagnosis of sarcoidosis was supported by the histologic findings in 121 patients (90.3%). In the 13 patients (9.7%) with a clinicoradiologic diagnosis of sarcoidosis, the stage distribution was as follows: five patients (38.5%) had stage I disease, five patients (38.5%) had stage II disease, one patient (7.7%) had stage III disease, and two patients (15.3%) had stage IV disease.

**Table 1:** Baseline Demographic and Clinical Characteristics of the 152 Patients With and Without Sarcoidosis Enrolled in the Study

Characteristic	Sarcoidosis Cohort (n = 134)	No Sarcoidosis Cohort (n = 18)	*P* Value
Age, y	52 (35-60)	50 (40-58)	.99
Female sex	76 (56.7)	6 (56.7)	.07
Ethnicity			1.00
White	125 (93.3)	17 (94.4)	
Black	6 (4.5)	1 (5.6)	
Other	3 (2.2)	0 (0.0)	
Smoking history			.04
Current	19 (14.2)	3 (16.7)	
Former	38 (28.4)	10 (55.6)	
Never	77 (57.4)	5 (27.8)	
Malignancy^a^	10 (7.5)	1 (5.6)	1.00
Sarcoidosis stage on CT scan imaging			.14
Lymphadenopathy	49 (36.6)	11 (61.1)	
Lymphadenopathy plus lung disease	79 (58.9)	6 (33.3)	
Lung disease	4 (3.0)	1 (5.6)	
Pulmonary fibrosis	2 (1.5)	0 (0.0)	
Airway abnormalities	64 (47.8)	7 (38.9)	.62
Pattern of AA^b^			
Nodularity	23 of 134 (17.2)	1 of 18 (5.6)	.18
Cobblestoning	8 of 134 (6.0)	1 of 18 (5.6)	.71
Plaque	19 of 134 (14.2)	0 of 18 (0.0)	.08
Thickening	9 of 134 (6.7)	3 of 18 (16.7)	.16
Increased vascularity	19 of 134 (14.2)	3 of 18 (16.7)	.50
Miscellaneous	1 of 134 (0.7)	1 of 18 (5.6)	.22
Final diagnosis of sarcoidosis			1.00
Clinical-radiologic-pathologic	121 (90.3)	13 (72.2)	
Clinicoradiologic	13 (9.7)	5 (27.8)	

Data are presented as No. (%) or median (interquartile range), unless otherwise indicated. AA = airway abnormality.

a Diagnosed in the 5 years preceding the patient’s enrollment in the present study.

b Total AA amounts to > 64 in the sarcoidosis cohort because in some patients more than one AA was identified and sampled.

HD videobronchoscopy found one or more areas of AA in 64 of 134 patients (47.8%) with sarcoidosis. On a per-patient analysis (Fig 3), nodularity (23/134 [17.2%]), plaque (19/134 [14.2%]), and increased vascularity (19/134 [14.2%]) were the most prevalent patterns. AAs were more prevalent in patients with sarcoidosis with parenchymal involvement on chest CT scan imaging than in those with isolated lymphadenopathy (52.9% vs 38.8%, respectively; *P* = .11).

Granulomas were detected in EBB specimens of 49 of 134 patients (36.6%) who finally received a diagnosis of sarcoidosis. AAs were significantly more prevalent in patients with than in those without nonnecrotizing granulomas in EBB specimens (67.4% vs 36.5%; *P* = .001). Moreover, parenchymal disease on CT scan imaging was significantly more common in patients with than in those without nonnecrotizing granulomas in EBB specimens (79.6% vs 54.1%; *P* = .003). The diagnostic yield of random EBB samples obtained from areas of normal-appearing airways was low (31/134 [23.1%]) and was not significantly different in patients with and without AAs (17/64 [26.6%] vs 14/70 [20%]; *P* = .37).

EBB was the only diagnostic bronchoscopic sampling method in 5 of 134 patients (3.7%). The other sampling methods yielding nonnecrotizing granulomas in our cohort were EBUS-TBNA in 103 patients, endoscopic ultrasound B with fine-needle aspiration in one patient, and transbronchial biopsy with standard forceps or with a cryoprobe in 14 patients.

Ninety-three mucosal areas were sampled in the 64 patients with AAs. In a per-lesion analysis, areas of cobblestoning (90%) and nodularity (58.6%) were associated with the highest detection rate for granulomas (Fig 3). AAs were seen more frequently in the upper lobes, but the diagnostic yield of EBB was not influenced by the location of the AA (e-Fig 2).

Parenchymal involvement on CT imaging (*P* = .003) and AAs (*P* = .001), especially areas of cobblestoning (*P* = .02) and of nodularity (*P* = .004), were observed more frequently in patients with positive EBB findings, whereas areas of increased vascularity were significantly more frequent (*P* < .0001) in patients with negative EBB findings (Table 2). In a multivariate analysis, the increased vascularity pattern was found to be an independent predictor of negative EBB results (e-Table 1).

**Table 2:** Demographic, Clinical, and Endoscopic Characteristics by EBB Results

Demographic and Clinical Variables^a^	Negative EBB Results (n = 85)	Positive EBB Results (n = 49)	*P* Value
Age, y	52 (39-60)	52 (34-60)	.68
Male sex	40 (47.1)	18 (36.7)	.25
Smoking history			.13
Current	12 (14.1)	7 (14.3)	
Former	29 (34.1)	9 (18.4)	
Never	44 (51.8)	33 (67.4)	
Ethnicity			1.00
White	79 (92.9)	46 (93.9)	
Black	4 (4.7)	2 (4.1)	
Other	2 (2.4)	1 (2.0)	
Current malignancy	8 (9.4)	2 (4.1)	.33
Sarcoidosis stage on CT scan imaging			**.003**
I	39 (45.9)	10 (20.4)	
II plus III plus IV	46 (54.1)	39 (79.6)	
AAs^b^	31 (36.5)	33 (67.4)	**.001**
AA pattern	(n = 43)	(n = 50)	
Cobblestoning	1 (2.3)	9 (18.0)	**.02**
Nodularity	7 (16.3)	22 (44.0)	**.004**
Plaque	11 (25.6)	10 (20.0)	.52
Thickening	5 (11.6)	5 (10.0)	.80
Increased vascularity	19 (44.2)	3 (6.0)	**< .0001**
Miscellaneous	0 (0.0)	1 (2.0)	.35

Data are presented as No. (%) or median (interquartile range), unless otherwise indicated. Boldface indicates statistical significance. AA = airway abnormality; EBB = endobronchial biopsy.

a Per-patient analysis.

b Per-lesion analysis.

The interobserver agreement between the operator performing the procedure and three operators from centers not enrolling patients for patterns of AAs was fair (Fleiss’ combined κ *=* 0.34). Among individual patterns, the agreement was substantial for areas of cobblestoning (Fleiss’ κ *=* 0.62); was moderate for areas of increased vascularity (Fleiss’ κ *=* 0.42); was fair for areas of plaque (Fleiss’ κ *=* 0.35), nodularity (Fleiss’ κ = 0.35), and thickening (Fleiss’ κ *=* 0.27); and was slight for miscellaneous (Fleiss’ κ *=* 0.07).

## Discussion

The present study evaluated HD videobronchoscopy for the detection of airway involvement in sarcoidosis and found tracheal or bronchial abnormalities, or both, in approximately one-half of patients (47.8%). This prevalence of AAs is much higher than that reported in the European studies on this matter. In a retrospective study conducted in Poland, Dziedzic et al^15^ performed EBB in 340 patients with suspected sarcoidosis and found AAs only in 52 patients (15.6%). Goktalay et al^16^ identified AAs in 20 of 59 patients (33.9%) with sarcoidosis, of whom seven had evidence of “extrinsic compression” rather than a mucosal abnormality. These figures provide preliminary evidence that HD videobronchoscopy might allow the identification of subtle airway changes that fiber-optic bronchoscopy or standard-definition videobronchoscopy are unable to detect.

In our study, the probability of detecting nonnecrotizing granulomas in EBB specimens was affected by the presence of AAs and of parenchymal involvement on CT scan imaging. Indeed, patients with AAs showed a significantly higher yield of EBB for the detection of nonnecrotizing granulomas than those with isolated lymphadenopathy. Although this finding is in line with the results of previous studies,^5^, ^6^, ^7^, ^8^, ^9^, ^10^^,^^16^ we tried for the first time to assess in a systematic fashion the prevalence and diagnostic yield associated with different patterns of AAs. Nodularity, plaque, and increased vascularity were the patterns detected more frequently, whereas the diagnostic yield of EBB was highest in specimens retrieved from areas of nodularity and cobblestoning. On the contrary, biopsy samples from areas of increased vascularity were associated with a very low diagnostic yield. Unfortunately, the overall interobserver agreement for patterns of AAs was only fair (κ = 0.34) and was substantial only for areas of cobblestoning (κ = 0.62). This aspect highlights the subjectivity of the categorization of the endoscopic findings, which is common to both neoplastic and nonneoplastic conditions.^23^^,^^24^

The detection of granulomas in EBB specimens was significantly higher in patients with parenchymal involvement on chest CT scan imaging than those with isolated lymphadenopathy in this cohort. We believe that two main factors should be considered to explain this finding. First, patients with parenchymal involvement showed a slightly higher prevalence of AAs, a finding that has been associated with an increased yield of EBB in most previous studies.^5^, ^6^, ^7^, ^8^, ^9^, ^10^^,^^16^ Another possible explanation would contemplate a different (ie, higher) burden of granulomas in the airways of patients with parenchymal involvement as compared with those with isolated lymphadenopathy on imaging studies. Notably, a different density of granulomas in different radiographic stages of sarcoidosis has been suggested to explain the higher yield observed with transbronchial lung biopsy in stages II and III,^25^^,^^26^ as well as with endosonography (EBUS-TBNA, endoscopic ultrasound B with fine-needle aspiration) in stage I.^27^^,^^28^ In previous studies, the detection rate of granulomas in EBB according to the Scadding stage assessed by chest radiography was variable. Most found a higher yield in stages characterized by the presence of parenchymal involvement (stages II-III),^9^^,^^10^^,^^12^^,^^14^^,^^16^^,^^17^ but others did not show any differences^21^ or even found a higher yield in stage I disease.^6^^,^^15^ This can be explained partially by the subjectivity and poor accuracy of the sarcoidosis stage classification based on chest radiography findings.^29^ Interestingly, in a large randomized study aimed at comparing EBUS-TBNA vs conventional TBNA in sarcoidosis, Gupta et al^9^ found that 33% of the patients classified as having stage I disease based on the chest radiography findings indeed showed parenchymal involvement on CT scan imaging.

The overall diagnostic yield of EBB guided by HD videobronchoscopy that we achieved (36.6%) is in line with or higher than that described in several studies,^8^^,^^9^^,^^11^, ^12^, ^13^^,^^15^^,^^16^^,^^18^, ^19^, ^20^, ^21^ but lower than that reported in others.^5^, ^6^, ^7^^,^^10^^,^^14^^,^^17^ However, in most previous studies, EBB was not performed in all patients with sarcoidosis diagnosed during the study period, leaving the presumption that EBB was performed mostly in patients with endobronchial changes, and leading to a selection bias. In a US study in which a 71% yield was obtained with EBB, only 56 of 150 patients who received a diagnosis of sarcoidosis during the study period underwent EBB; the authors explicitly state, “EBB were taken primarily from patients with abnormal bronchoscopic findings (52 of 56 patients).”^5^ Furthermore, the high yield of EBB (57%-71%) was achieved in US sarcoidosis populations,^5^, ^6^, ^7^ which included a high proportion of African American patients and was never confirmed by studies conducted in Asia (6%-49%),^8^, ^9^, ^10^, ^11^^,^^20^^,^^30^ Europe (18%-45%),^14^, ^15^, ^16^, ^17^, ^18^, ^19^ or Oceania (27%-28%),^13^^,^^21^ a result that suggests a key role played by ethnicity.

In studies aimed at evaluating the added value of EBB when combined with other sampling methods (eg, BAL, transbronchial biopsy, conventional transbronchial needle aspiration), a 2% to 21% increase in diagnostic yield was reported.^5^, ^6^, ^7^, ^8^, ^9^, ^10^, ^11^, ^12^, ^13^, ^14^, ^15^ Notably, EBB proved more useful in studies carried out before the availability of endosonography, a method that rapidly has become the first-step diagnostic procedure owing to its high diagnostic success and safety.^25^^,^^31^, ^32^, ^33^ Therefore, it is unsurprising that the added value of EBB in our study, in which endosonography was used along with EBB in most patients, was only 3.7%.

Recently, pilot studies assessed the possible role of image-enhancing technologies in the detection of AAs in sarcoidosis. In a case report, Hakim et al^34^ described the usefulness of narrow band imaging, an optical image-enhancing technology largely used for the detection of premalignant and early malignant mucosal lesions, in the identification and sampling of small hypovascular sarcoidosis-related nodular lesions. More recently, Dhooria et al^30^ demonstrated a higher sensitivity of narrow band imaging when compared with conventional white light bronchoscopy to detect AAs in sarcoidosis in a small retrospective study. A large randomized trial (ClinicalTrials.gov Identifier: NCT05311150) aimed at comparing the diagnostic yield of EBB guided by narrow band imaging or white light bronchoscopy for the diagnosis of endobronchial sarcoidosis currently is ongoing. In the present study, we used the i-scan, a digital image enhancement technology that provides the physician with an enhanced view of the mucosal structures (i-scan 1) and the vascular patterns (i-scan 2), supporting early detection, characterization, and demarcation.^24^^,^^35^ We found the i-scan technology useful because it increased our confidence in the identification and characterization of subtle mucosal changes (Video 1), especially small hypovascular lesions (Video 2), as compared with the standard HD method. However, the extent to which this technology may increase the detection of subtle airway changes or characterize their pattern better needs to be assessed rigorously in future studies using the i-scan as a predefined end point.

The prospective international multicenter design, the large sample size, and the systematic assessment of AAs categorized as different patterns defined a priori are the strengths of our study. However, some limitations need to be acknowledged. First, the study population had specific demographic (large prevalence of White Europeans), clinical (approximately two-thirds of the patients with parenchymal involvement resulting from sarcoidosis), and endoscopic (47.8% with AAs) features. Therefore, the reproducibility of our results in populations with different characteristics is uncertain. Second, a formal sample size calculation was not performed because no previous study had explored the yield of EBB guided by HD videobronchoscopy for the diagnosis of tracheobronchial sarcoidosis, and the yield of EBB in studies that used white light bronchoscopy or standard definition videobronchoscopy was widely variable. Finally, because of the referral base of our bronchoscopy practices, the prevalence of extrathoracic involvement resulting from sarcoidosis was not captured in this study. However, we believe that although this is meaningful information, it does not affect the relevance of our study’s results.

## Interpretation

To our knowledge, the present study provided the first appraisal of prevalence and pattern of AAs, as well as of the diagnostic yield of EBB guided by HD videobronchoscopy in patients with suspected sarcoidosis. Given its simple technical performance, low morbidity, and nonnegligible diagnostic yield for the detection of granulomas (33%-90%), we suggest that EBB be used in patients with clinicoradiologic suspicion of sarcoidosis featuring any AA other than the increased vascularity pattern.

## Funding/Support

The authors have reported to *CHEST* that no funding was received for this study.

## Financial/Nonfinancial Disclosures

The authors have reported to *CHEST* the following: I. S. has received honoraria for lectures, fees for consultation, and support for training courses from Pentax outside the submitted work. J. T. A. has received educational support for courses from Pentax outside the submitted work. P. C. has received honoraria for lectures from Pentax outside the submitted work. I. V. has received honoraria for lectures from Pentax outside the submitted work. R. T. has received honoraria for lectures from Pentax outside the submitted work. None declared (V. L., T. K., M. F., K. M., D. F., S. M., A. C., L. A. S., M. P., G. S.).

## Supplementary Data

e-Online Data
