# Supplementary material for: High-Definition Videobronchoscopy for the Diagnosis of Airway Involvement in Sarcoidosis: The Enhance Sarcoidosis Multicenter Study
Source: Chest. 2023 Apr 28;164(5):1243–52. doi: 10.1016/j.chest.2023.04.034 (PMC10635836; doi:10.1016/j.chest.2023.04.034)

**HIGH DEFINITION VIDEOBRONCHOSCOPY FOR THE DIAGNOSIS OF AIRWAY INVOLVEMENT IN SARCOIDOSIS: THE ENHANCE SARCOIDOSIS MULTICENTER STUDY**

Vanina Livi, Ilya Sivokozov, Jouke T. Annema, Piero Candoli, Igor Vasilev, Tess Kramer, Marco Ferrari, Madan Karan M, David Fielding, Septimiu Murgu, Alessandra Cancellieri, Mariangela Puci, Giovanni Sotgiu, and Rocco Trisolini.

**SUPPLEMENTARY MATERIAL**

**METHODS**

**HD videobronchoscopy settings**

In order to guarantee the reproducibility of the results, the following settings of the EPK 3000 Defina processor were used in the centers involved in the study to perform HD bronchoscopy, i-scan 1 and i-scan 2:

- *HD broncoscopy*: Noise reduction=High; D-range expansion=Low; Contrast=High; Freeze scan=Middle; Max brightness level=Normal; White balance=Normal; Film counter type=1-99; Image size=Full
- *scan 1:* Brightness=0; Red=0; Blue=0; IRIS:Average/Peak=Average; Noise Reduction=High; Enhancement=+2; SE=+5; CE=Off; TE=Off; D-range expansion=Low.
- *scan 2:* Brightness=+1; Red=0; Blue=0; IRIS:Average/Peak=Average; Noise Reduction=High; Enhancement=+2; SE=+4; CE=Off; TE=c; D-range expansion=Low.

**e-Table 1. Logistic regression to assess relationship between anthropometric, epidemiological, clinical characteristic, and endobronchial biopsy.**

| **Variables** | **Univariate analysis** | | | | **Multivariate analysis** | |
| --- | --- | --- | --- | --- | --- | --- |
|  | **OR 95% CI** | **p-value** | | | **OR 95% CI** | **p-value** |
| **Age, years** | 0.99 (0.97-1.02) | | 0.56 | 0.98 (0.93-1.03) | | 0.43 |
| **Males** | 0.65 (0.32-1.34) | | 0.25 | 0.77 (0.23-2.64) | | 0.68 |
| **Smoking habit**  Never  Current  Former | Ref.  0.78 (0.28-2.19)  0.41 (0.17-0.99) | | Ref.  0.63  0.05 | Ref.  -  - | | Ref.  -  - |
| **Ethnicity**  Black and Other  White | Ref.  1.17 (0.28-4.88) | | Ref.  0.84 | Ref.  - | | Ref.  - |
| **Current malignancy** | 0.41 (0.08-2.01) | | 0.27 | - | | - |
| **Sarcoidosis stage on CT**  Parenchymal involvement  No parenchymal involvement | Ref.  0.30 (0.13-0.68) | | Ref.  0.004 | Ref.  0.44 (0.11-1.84) | | Ref.  0.26 |
| **Airway abnormalities** | 3.59 (1.71-7.55) | | 0.001 | - | | - |
| **Pattern of airway abnormality**  Cobblestoning  Nodularity  Plaque  Thickening  Increased vascularity | 8.08 (0.93-70.04)  3.80 (1.03-11.05)  0.93 (0.26-3.25)  0.93 (0.21-4.10)  0.07 (0.01-0.34) | | 0.06  0.04  0.90  0.93  0.001 | -  2.21 (0.52-9.30)  -  -  0.10 (0.02-0.56) | | -  0.28  -  -  0.008 |

Hosmer_Lemeshow p-value= 0.16

Airway abnormalities excluded because of collinearity

**# Reference level: presence VS. absence of single specific pattern**

**e-Figure 1**


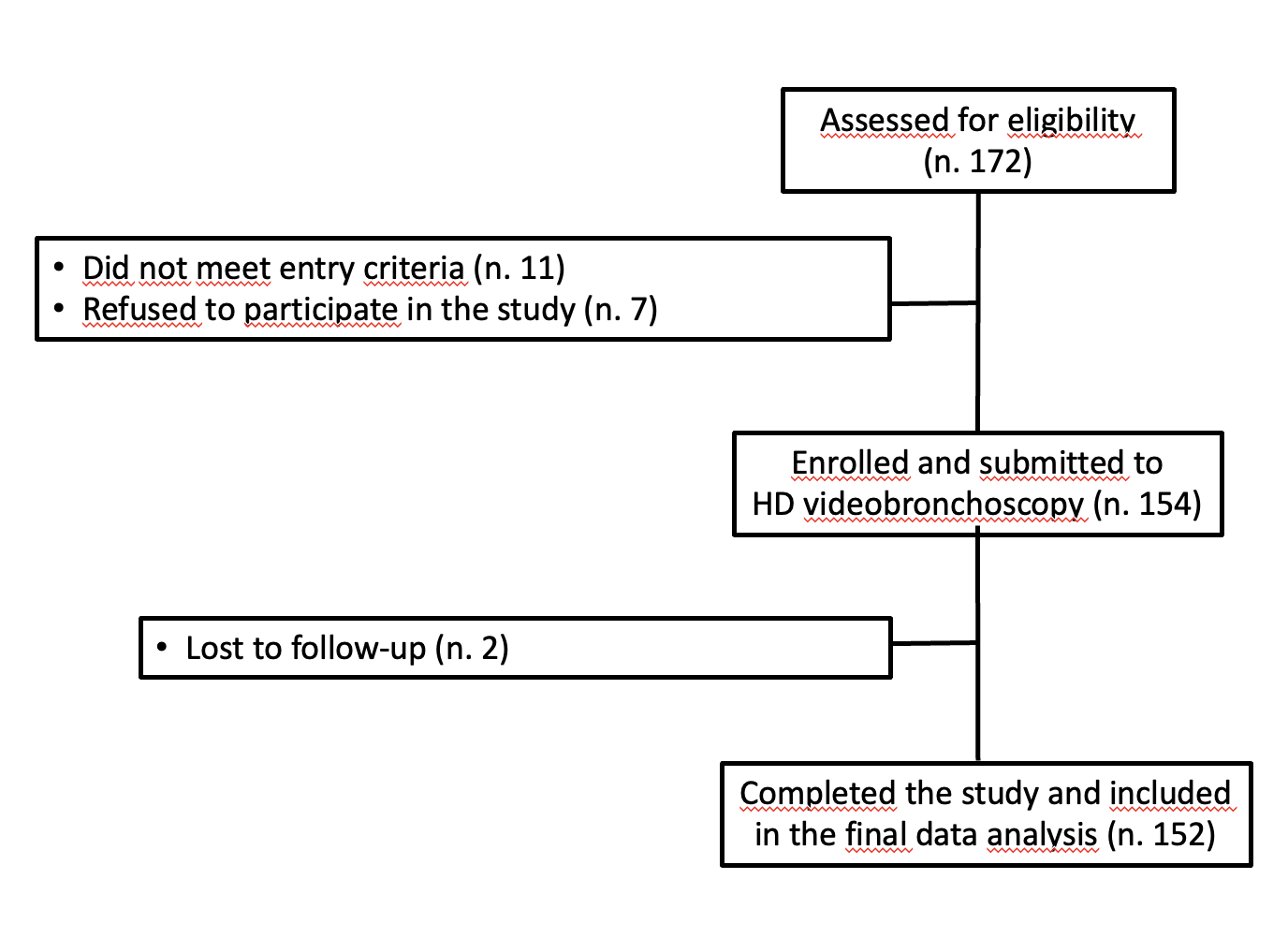


**e-Figure 2**


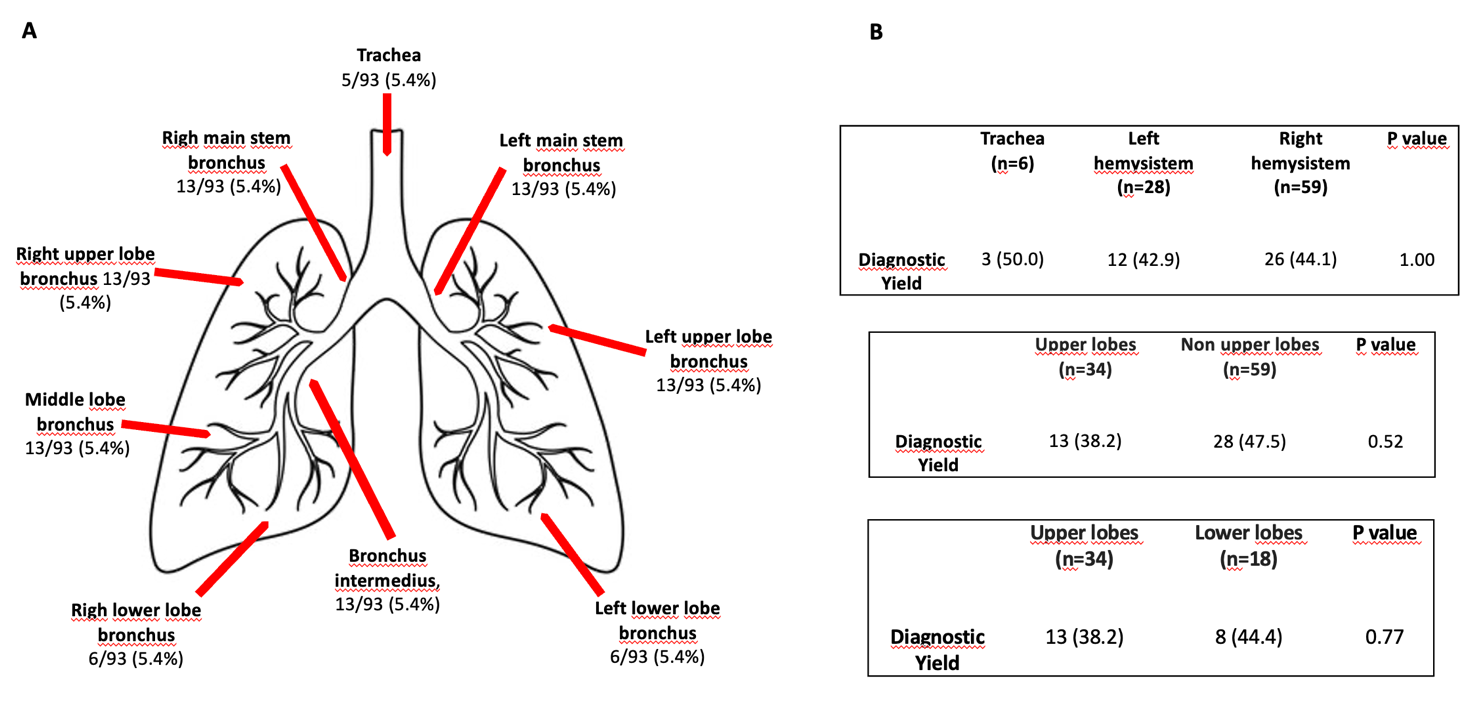

Supplement: e-Online Data [file mmc1.docx]
